# Transdermal Evaporation Drug Delivery System: Concept to Commercial Products

**DOI:** 10.15171/apb.2018.063

**Published:** 2018-11-29

**Authors:** Rabinarayan Parhi, Suryakanta Swain

**Affiliations:** ^1^GITAM Institute of Pharmacy, GITAM (Deemed to be University), Gandhi Nagar Campus, Rushikonda, Visakhapatnam-530045, Andhra Pradesh, India.; ^2^Southern Institute of Medical Sciences, College of Pharmacy, Department of Pharmaceutics, SIMS Group of Institutions, Mangaldas Nagar, Vijyawada Road, Guntur-522 001, Andhra Pradesh, India.

**Keywords:** Transdermal, Supersaturation, Stratum corneum, Crystallization, Thermodynamic activity

## Abstract

Since two decades or so transdermal route established itself as better alternative to traditional oral route. This is possible due to continuous innovations in transdermal drug delivery (TDD), which not only enables researchers from academia and industry to successfully develop and launch many new pharmaceuticals but also allow to include new classes of drugs that can be developed into transdermal formulations. These successes are achieved due to the use of novel techniques based on either physical or chemical approaches. However, both of these techniques suffer due to their own disadvantages. Comparatively, a simple method of supersaturation to enhance drug permeation across skin has created a new wave of interest. Even though the application supersaturated principle in topical and TDD has been used from 1960s, but proper control of drug release and formation of stable supersaturated states has been the core of intense research in the last decade. Out of various methods used to get supersaturated system, evaporation method is considered as most efficient and practically feasible for TDD. Therefore, in this review concept of supersaturation, selection of solvent system and the mechanism of inhibition of crystallization are discussed. Application of evaporation systems in the development of transdermal formulations such as solutions, semisolids and metered dose therapeutic systems (MDTS) and the commercial evaporative systems are also discussed in this review.

## Introduction

Delivery of drug across the skin i.e., transdermal drug delivery (TDD) offer an alternative route to traditional oral and parenteral routes with many distinct advantages such as avoidance of first pass metabolism by liver and gastro-intestinal tract, improves patient compliance by reducing the frequency of drug administration, minimizes the side effect of drug caused due to temporary overdose, steadier drug plasma concentration, non-invasive drug delivery i.e., without any needle-phobia etc.^1-3^ Common dosage forms applied topically to provide systemic effect are creams, ointments, gels and patches.^4^ It is obviously difficult to sustain the effect of drugs present in creams and ointments for the considerable period of time after application because of every possibility of their removal from the skin surface as a result of movement, contact and wetting.^5^

Presently, patch and gel formulations are widely used in clinical settings owning to their increasing number of available formulations commercially such as Transderm Scop, Nitroderm, Nitrodisc etc. as patches and Androgels, Androderm etc. as gel. Contrary to creams and ointments, gels with bioadhesive polymer (s) provide optimum contact times for the drug absorption.^6,7^ Gel formulation has the disadvantages such as to achieve desired plasma levels of drugs it has to apply over large surface area and there is always possibility of gel being transferred to female partners thereby further reducing the available drug amount for absorption. This particular problem can be avoided by using approved transdermal patches. However, local skin reactions such as redness, irritation, blistering and microbial proliferation caused by occlusion most often associated with patch formulation, which significantly reduces patient compliance as well as acceptance.^8-12^ Not only that, manufacturing of multi-component systems such as patch has always been a challenge to formulation scientist. In addition, instability of patches especially drug crystallization on long storage is being a herculean task to be solved.

Skin is considered as largest organ of our body as it accounting for 10% of body mass. Despite of large surface area available for drug transport across the skin, penetration of drug into skin is limited by stratum corneum (SC), the uppermost layer of skin.^13^ Manipulation of SC barrier can be carried out either by chemical^13,14^ and several physical approaches^15-18^ in order to improve drug permeation across the skin. Chemical approach using penetration enhancers (PEs) have definitive advantages such as they provide reversible damage to the skin, painless administration, give the flexibility to formulator to alter the formulation composition according to the need. Despite all this chemical PEs such as surfactants and organic solvents are resulting in different skin ailments such as stinging, burning, erythema and contact urticaria.^19^ Furthermore, there is no report on the long-term effect of PEs on the SC.^20^ No doubt that various physical techniques including iontophoresis, phonophoresis and more recently microneedle technique have tremendous potential in enhancing the drug permeation across the skin. But, the main concern with above techniques is the pain at the administration site. Furthermore, an additional step of microneedle sterilization increases the cost effectiveness of the system.^21^ In this scenario, supersaturation technique seems to be one of the attractive alternatives.

## Supersaturation

**Figure 1:**
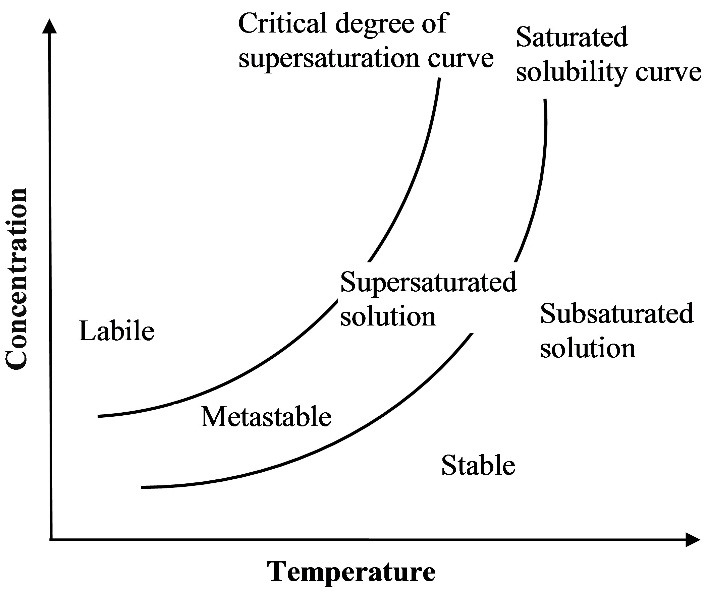
Diagram showing the critical degree of supersaturation and the different stability states of supersaturated systems.

The concept of supersaturated systems originated from the crystallization theory, in which the formation of supersaturated solution is the first step of crystallization. A supersatuarated solution can be defined as the one which contains more amount of dissolved solute than it would normally contain in a saturated solution at a constant temperature. As supersaturated systems are considered as thermodynamically unstable (labile state), in most of the cases, there is spontaneous formation of crystals (Figure 1). Metastable is a state in which solute material remains in the supersaturated solution without crystallization. This state is maintained until any foreign solid particles are added intentionally/accidentally or the system is subjected to external forces (e.g., ultrasound). Thus, the line between labile and metastable states is mentioned as critical degree of saturation.^22^

**Figure 2:**
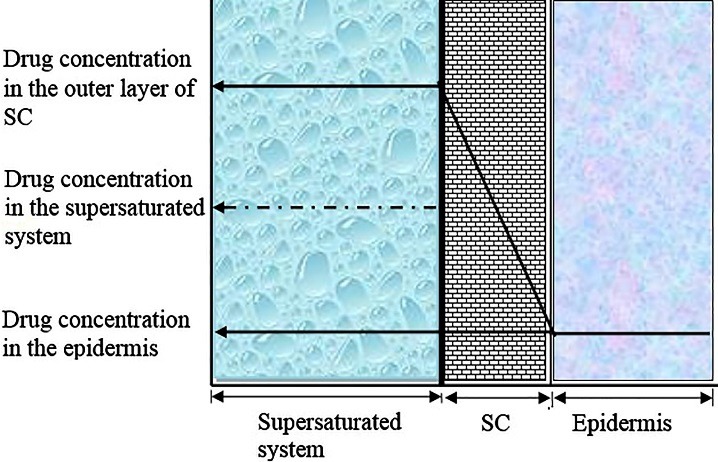
Schematic profile of steady-state diffusion across the skin (Modified from reference 22).

Even though supersaturation concept is old concept, the benefits of it to transdermal/topical drug delivery were first recognized by Higuchi in 1960.^23^ According to him the rate-controlling step to percutaneous absorption of drug from supersaturated system was resides in the outer layer of SC (Figure 2). Based on the definition of partition coefficient, when a vehicle is saturated with a drug applied on the skin surface, then it must lead to saturation of SC with the same drug. If excess than this saturation level of drug present in the vehicle, there will be increase in drug amount in the SC proportionately. Hence, it leads to penetration enhancement of drug in the same level of degree of supersaturation.^22^ Later on, it was understood that above concept was also based on potential concentration gradient. Concentration gradient of a supersaturated system of drug is significantly higher than that of its saturated system across the skin surface. Therefore, a similar rise in concentration of the drug in the outer layer of SC in order to maintained equilibrium at least for a short period of time. This is only feasible if the lipids in the SC permit the drug to remain in higher concentration/activity level i.e., antinucleate agents.^22^

Broadly, there are five methods to obtain supersaturated systems:^24,25^

1. Heating and cooling
2. Evaporation of the solvent
3. By adiabatic evaporation i.e., cooling plus evaporation
4. Salting out i.e., addition of a substance to a solution that reduces the solubility of the primary solute
5. Formation of a new compound, due to the reaction between two or more solutes, which is poorly soluble compared to initial solute.

In addition, sometimes change in pH bring about change in solubility and resulted in supersaturated solution. Present review mainly focused on the formation of supersaturated system by evaporation method.

### Advantages

1. Evaporative systems provide passive and nonocclusive delivery of drugs.^26^ Therefore, this system is well tolerated and also demonstrates very low skin irritation rates.^27^
2. Compared to physical technologies they are having simple working mechanism and more importantly they provide non-invasive drug delivery. Similarly these systems less likely to provoke skin irritation when compared with chemical PEs.^28^
3. These systems are inexpensive and provide penetration enhancement that is specific to the drug without disturbing the integrity of the SC.^22,29^
4. These formulations are fast drying products where the volatile component provides uniform distribution of drug on the skin surface with minimum vehicle deposition; thereby it reduces the uneasiness of patient.^30^
5. Manufacturing and scale-up of these systems are much less tedious than patch formulations.^27^
6. With single application of these systems, a sustained steady-state serum drug level can be maintained over 2-4 days.^31^
7. These systems offers ease of application, specifically when a metered dose transdermal system (MDTS) is used.
8. MDTS make sure that only pre-determined dose is administered which is aesthetically appealing and also able to eliminate concerns of residual drug associated with transdermal patches.^30^
9. In these systems, the patient have the flexibility to vary delivery dose according to the situation as it is packaged in a multi-dose container.^31^

### Disadvantages

1. Like other transdermal dosage forms, these systems have relative low bioavailability compared to oral and parenteral dosage forms.^27^
2. Selection of solvent system is always posing a challenge to formulation scientist as the thermodynamic effect of drug depends on the solvent system selected.
3. Active ingredients for this technique would require a suitable drug-response profile.^31^
4. Supersaturated formulations are inheritently thermodynamically unstable and may lead to drug crystallization. In most of the case this problem can be overcome with antinucleate polymers which either inhibits nucleus formation or retard the crystallization.^20,28,29^

## Selection of vehicle

The solvent in which a drug is present can increase the drug permeation through the skin by either ways: (i) by increasing the thermodynamic activity of the drug or (ii) altering the barrier property of the SC. Thermodynamic activity of a drug mainly depends on the solvent in which it is solubilized. Therefore, the selection of solvent is very crucial for enhanced permeation of drug across the skin. It has to keep in mind that maximization of thermodynamic activity is essential to achieve higher concentration of drug in the SC. The flux is related to thermodynamic activity as expressed in the following equation:

$$\frac{dm}{dt}=J(flux)=\frac{\alpha D}{\gamma h}$$

Where α is the thermodynamic activity of the drug in the donor formulation, γ is effective activity coefficient in the skin barrier, D is diffusivity and h is the thickness of the membrane. So, more the thermodynamic activity of a drug in a formulation higher will be the flux as the escaping tendency of the drug from the vehicle is at the peak.^32^ Furthermore, thermodynamic activity of a drug in a solvent is proportional to the ratio of its concentration to solubility in that vehicle. Thus, more the solubility of drug in the vehicle less will be the thermodynamic activity. There are two approaches employed to study the effect of different vehicle/solvent on the permeation of a drug across the skin. In the first approach, a fixed concentration of a drug is prepared in different solvents. This method is helping in selecting appropriate solvent with higher thermodynamic activity for a drug whose concentration is already fixed. In second approach saturated solutions of a drug in different solvents are prepared (solute activity is considered as unity). This approach should give equal flux provided the solvents are not having skin barrier alteration potential.^33^ Above equation also illustrates that different formulations of any particular drug at equal thermodynamic activity will provide equal penetration provided excipients in the formulation do not influence any property of drug. Implication of this is that by selecting appropriate formulation drug loading can be minimized by maintaining equal thermodynamic activity, thereby delivering equal amount of drug. For example; 0.1% hydrocortisone gel (Dioderm®) is clinically equivalent to 1% hydrocortisone cream, BP. Though both are having different concentrations of drug, but the thermodynamic activity is equal. Therefore in the successful development of transdermal/topical supersaturated systems it is essential to study the flux of various vehicles/solvents at fixed drug concentration as well as at saturation level.^34^

## Mechanism of penetration enhancement by evaporation means of supersaturation

Any subsaturated or saturated solution can be converted to supersaturated one by evaporating the solvent as the same amount of solute will be present in gradually decreasing amount of solvent. This is more pronounced if any volatile component/solvent is present the solution. This principle is explored to develop transdermal and topical formulations in order to improve thermodynamic activity of active ingredient and thereby enhance its penetration into/across the skin.^35^ For instance, an active ingredient not present in supersaturated level in any formulation, but when applied on the skin surface volatile solvent evaporates, thereby reducing solubility in the residual amount of solvent. The reduction of solvent amount lead to the formation of supersaturated state of the active ingredient. Certain topical formulations in the market (e.g., lotions) are forming supersaturated states, resulting in the penetration enhancement of active ingredients.

Basically, evaporation systems consisting of two components: drug and solvent mixture. The solvent mixture is composed of both volatile and nonvolatile solvents. Here the pre-requisite is that the selected drug must soluble in both the solvents. The volatile component ensures that the dose can be administered in a precise (volume per area and easy spreading of applied formulation) and reproducible manner. More importantly, it increases the concentration of drug in the formulation.^36^ Non-volatile solvent prevents the precipitation of drug from the solution, when the volatile counterpart gets evaporated. Non-volatile solvent selected should have higher partitioning property. This physicochemical property not only helps in partitioning of solvent and drug into the SC, but also serving to disrupt the ordered intercellular lipids and enhance permeation.^31^ Upon application of an evaporation delivery system on the skin surface, it creates a depot system in the SC from which the drug can be absorbed into systemic circulation for a longer period of time. Therefore, evaporation delivery systems ensure sustained plasma drug concentration for longer period of time.

## Mechanism to inhibits or retard crystallization

It is mentioned in the earlier section that the instability is one of the major limitations for the supersaturated systems. The instability is due to rapid crystallization of drug when the volatile component is getting evaporated immediately after the application of these formulations. Formation of crystals not only reduces the initial high activity of the drug but also gives an irregular plasma drug concentration. Therefore, stabilization of thermodynamically unstable states is important step in the development of successful supersaturated systems with desired permeation enhancement across the skin. It is well known that the instability of supersaturated system lead to establish the foundation for crystallization i.e. formation of nucleus, which subsequently resulting in crystal growth. In addition, there are many external factors such as type of solvent, temperature variation (based on the heat of solution), impurities, pH and hydrodynamics. Furthermore, the nucleation time and the time needed to form critical sized nuclei, from where the crystal growth can take place, decreases with the increase in degree of saturation (DS). In order to stabilize the system, one or more of these factors are often used to control both the nucleus formation and its growth.^29,37^

When a supersaturated solution of a drug is applied on the skin surface, whether it contains PEs or not, the concentration of drug in the upper layer of SC is also supersaturated. This may lead to crystal formation in the upper layer of SC, which can reduce the rate of drug permeation into systemic circulation. In one study it was found that penetration enhancement of piroxicam was increased which may be due to antinucleating ability of the intercellular lipids of SC.^38^ One of the very widely used methods is to selectively use antinucleant polymers to stabilize the unstable supersaturated systems.^20,39-41^, Polymers such as hydroxypropyl methylcellulose (HPMC), methylcellulose (MC) and polyvinyl pyrrolidone (PVP) are already tested as antinucleate polymers.^29^ Second method to prevent nucleus formation and crystal growth is to use tailor made additives (impurities). The inhibition of crystal growth of paracetamol was succefully performed by the addition of para-acetoxy acetanilide.^42^ It was observed that by the addition of small quantities of fatty acid as additives was able alter the physiochemical properties of adipic acid tremendously by incorporating into crystal.^43,44^ In addition to above techniques, a stable supersaturated solution is achievable with low DS compared to higher one. This is observed with ibuprofen; with higher DS the solution was converted to transulescent demonstrating the presence of ibuprofen micro-crystal whereas at lower DS the solution was found to be clear one.^41^

## Mathematical modeling

Flux of a permeant across a membrane from a saturated solution is constant and independent of its concentration. Therefore, under normal conditions, the flux of a drug is limited by its saturated solubility. The flux of drugs across a membrane at the steady state is governed by Fick’s first law of diffusion and expressed as:

$$J\;=\frac{DAK}{h(Cv-Cr)}$$

Where J is flux, D is the diffusion coefficient, K is partition coefficient between membrane and vehicle, h is the diffusional path length, and Cv and Cr are drug concentration in the solution/formulation and in receptor phase, respectively. Under sink condition (Cv≥0.1Cr), Cr is considered as negligible; therefore (Cv-Cr) term is usually approximated as Cv.^22,20^

## Applications of evaporation based supersaturated principle

Supersaturated principle based on evaporation is mainly applied in transdermal/topical drug delivery and the formulations in which this concept is incorporated are topical solutions and semisolids and spray. The latter formulation is now being widely used in transporting drug across skin. In addition there are many commercial products available based on spray technique. A list of drugs, solvent systems, polymers with their references are presented in Table 1.

### Solutions and semisolids

Poulsen *et al.* performed a series of experiments in order to find out the correct and optimum vehicle for the release of fluocinolone acetonide and its acetate derivatives from a gel comprising of propylene glycol (PG), water and isopropyl myristate (IPM) as vehicle. The solubility and concentration of the drug in the vehicle and partition coefficient between the vehicle and IPM were found to be determining factors for the release of drug from the prepared gel.^45^

Coldman *et al.* studied the potential of supersaturated systems of fluocinolone acetonide and its acetate esters and compared their penetration enhancement with that occluded formulations. The volatile component was isopropanol and non-volatile components were IPM and PG. After the application of above formulations, the volatile component gets evaporated leading to the formation of supersaturated state of the drug, thereby increased the penetration of drug. In the comparison between occluded and unoccluded systems, the unoccluded formulations were found to show higher penetration.^46^

Theeuwes *et al.* developed supersaturated solution of hydrocortisone using volatile/non-volatile co-solvent system. Acetone and water were volatile and non-volatile components, respectively. In the first step, mixture of acetone and water was prepared and then hydrocortisone was dissolved in it. After the application of above drug mixture on ethylene-vinyl acetate (EVA) membrane, acetone evaporates leaving supersaturated solution of hydrocortisone. This resulted in the 3.9 fold increase in saturation value, giving a proportional increase in flux.^47^

Kondo and Sugimoto investigated the diffusion of nifedipine across EVA copolymer membrane from different combinations of solvents such as IPM, PG and acetone. They observed that the initial flux was found to be higher than the steady-state fluxes. This was attributed to the initial evaporation of volatile solvent resulted in the formation of a saturated solution, thereafter the drug was crystallized out of the solution. The crystallization of drug was prevented by the incorporation of an antinucleate polymer into IPM:PG:acetone vehicle thereby enhancing the flux by 5-fold.^48^

In another study, the diffusion of nifedipine was studied across EVA copolymer membrane (*in-vitro*) and excised male Wistar rat skin (*in-vivo*) from vehicle consisting of ethanol (volatile component) and diethyl sabacate (nonvolatile component). With both *in-vitro* and *in-vivo* studies, highest diffusion was exhibited by ethanol:diethyl sebacate in the 75:25 ratio. Furthermore, the same combination showed 1.75 times of saturation after complete evaporation of the ethanol.^49^ The same group of researchers investigated the *in-vivo* penetration of nifedipine from range of volatile (acetone)/ non-volatile solvent (PG and IPM) combinations across male Wister rat skin. The initial plasma concentrations of drug were higher from both binary mixture (PG:acetone and IPM: acetone) and ternary mixture (PG:IPM:acetone) due to the formation of supersaturated solution after the evaporation of acetone. The plasma concentrations of drug were decreased in the later stage due to precipitation of drug, but upon incorporation of an antinucleate polymer to the vehicles, higher plasma levels were observed. Furthermore, ternary systems exhibited greater degree of penetration compared to binary systems.^50^

Chiang *et al.* used ethanol as volatile component and PG and water as non-volatile components to enhance the flux of minoxidil through human cadaver skin. The evaporation of the volatile component mostly happened within 30 min of application at room temperature. The flux value was increased with increasing concentration up to a point where the crystallization of drug started. There were no statistically significant differences of minoxidil flux when PG content in the vehicle was varied at the expense of ethanol without changing drug concentration.^51^

McDaid and Deasy attempted to enhance the thermodynamic gradient of nifedipine across the hairless mouse skin by the use of evaporation system comprising of ethanol: butanone: water in the ratio of 18.75:37.5:43.75% v/v. The flux of nifedipine was enhanced using evaporation system compared to aqueous suspension by 1330, 2897 and 172-fold using three different types of membrane such as hairless mouse skin, Celgard 2400 and EVA 987192. They attributed the above results could be due to interaction between the solvent system and various membranes rather than the increased drug solubility in volatile/non-volatile vehicle.^52^

Pellett *et al.* prepared supersaturated solutions of piroxicam in a 40:60 (v/v) PG/water co-solvent mixture and studied the effect of different DS on the permeation of piroxicam across silicon membrane and full-thickness human skin. In general, there was a linear correlation between the flux and DS for the supersaturated systems, but flux values across human skin at 0.5 and 1 DS were found to be similar. This was partly due to difference in solubility of the drug at different donor phase temperatures. It was observed that solutions up to 4 DS were stable up to 16 h. It was also observed that addition of HPMC to the solution prevented the formation of hydrate form of piroxicam, which was less soluble than its anhydrous form in the mixture of aqueous solvent.^20^

Pellett *et al.* investigated the *in-vitro* permeation of piroxicam from supersaturated solutions up to 4 DS across human skin and the amount of permeant in SC was estimated by tape stripping method. It was observed that there was a linear relationship between the DS and the amount of piroxicam in the SC with R^2^ value of 0.970. In addition, piroxicam amount in viable layers of the skin also increased with the increase in DS. Above result was attributed to the antinucleating ability of intercellular lipids of the SC that inhibits or retards the formation of drug crystals in SC.^38^

Cho and Choi tested various vehicles in order to evaluate their influence on the permeation rate of ketoprofen across mouse skin. Vehicles such as octanol, ethanol, and PG/Oleyl alcohol (OA) mixture exhibited the highest flux of 30 µg/cm^2^/h from 5mg/ml solution. However, there was no correlation between the solubility of ketoprofen in those solvents and its permeation rate. This enhancement of ketoprofen was attributed to the change in barrier property of mouse skin and/or carrier mechanism of the vehicles used. When the effect of vehicles on the percutaneous absorption of ketoprofen from acrylic pressure sensitive adhesive (PSA), oleic acid exhibited marginally higher flux of 2 µg/cm^2^/h compared to other solvents tested.^34^

Fang *et al.* developed aqueous ethanolic vehicle as evaporative system to enhance the delivery of sodium nonivamide acetate across rat skin. To stop the crystallization of drug as the volatile component evaporates on the skin surface, a range of potential antinucleant polymers were investigated. The delivery of drug was increased rapidly over the first 12 h thereafter a plateau up to 72 h. It was attributed to: (i) precipitation of drug on the skin surface and (ii) may be the crystallization within the skin membrane itself limited the continued increased in permeation. Among all, MC and hydroxypropyl cellulose (HPC) were found to be the best antinucleant polymers.^53^

Iervolino *et al.* developed supersaturated solutions of ibuprofen ranging from 0.5 to 5 DS in 40:60 (v/v) PG/water mixtures in the absence and presence of additives such as HPMC and 2-hydroxypropyl-β-cyclodextrin (CD) and investigated ibuprofen permeation across human epidermis from supersaturated solution. Supersatuarated solutions of drug showed significant flux enhancement compared saturated solutions of drug. In the absence of HPMC, the flux of ibuprofen was found to be below per expected for 4 and 5 DS. In the absence of additives, the flux was lower than expected for 4 and 5 DS whereas the addition of HPMC enhanced the flux in proportional to the DS because of stabilization of the supersaturated solutions. The stabilization is due to the formation of hydrogen bond between HPMC and ibuprofen which inhibit the growth of ibuprofen crystal. With CD at 2 and 3 DS, the flux of drug was found to be less compared not only to supersaturated solution with HPMC but also to saturated solutions due to the formation of ibuprofen/CD inclusion complex. But at 5 DS a higher enhancement of flux was observed indicating that CD might act as PE.^29^

Moser *et al.* prepared different supersaturated formulations of a lavendustin derivative (LAP) employing techniques such as (i) mixed co-solvent (e.g., PG-water, PEG-water, IPM-silicone oil), (ii) method of solvent evaporation such as ethanol-PG, and (iii) the method of dissolving the drug by heating. Formulations at 1 DS showed comparable permeation across the pig skin. Whereas formulations at 2 DS resulted in 2-fold increase in drug permeation and were independent of the type and composition of the vehicles, method of preparation and absolute concentration of the drug. Although supersatuarated solutions at 2 DS such as PG-water and ethanol-PG exhibited significant increase in LAP transport compared to 1 DS, but the later formulation showed highest increase (2.2-fold) in LAP transport.^54^

Santosh *et al.* prepared supersaturated solution of oxybutynin by solvent evaporation technique with solvent/vehicle such as ethanol, PG and octyl salicylate (OS) and studied the effect of different DS and solvents on the permeation of oxybutynin across human abdominal skin. There was an insignificant difference in oxybutynin permeation following the application of 25% PG containing formulations with 1, 2, and 5 DS. This result indicated that permeation enhancement could not be achieved employing supersaturated PG formulations. When 25% OS formulations with different DS were tested, 5 DS formulation showed lowest permeation (Q24h, 0.064±0.028 µm/cm^2^) whereas with 50% OS formulations at 1 DS exhibited lowest permeation (Q24h, 0.052±0.008 µm/cm^2^). Furthermore there were decrease in drug permeation with increase in DS of drug for OS containg formulations, which was attributed to decrease in both drug crystallization and solvent activity (OS uptake) with increase in DS.^35^

Wang *et al.* developed transdermal evaporation delivery system of praziquantel. They have used ethanol as volatile component and various non-volatile solvents such as ethylene glycol monophenyl ether (EGPE), tetrahydrofuran, 1, 4-dioxane, oleic acid and dimethyl sulfoxide (DMSO) and studied the solubility of drug in those solvents at different temperatures. Among all, EGPE was selected as non-volatile component as the drug solubility was found to be highest (more than 400 mg/mL) when it is mixed with ethanol (50% w/w) at 32ºC. The serum drug concentration was found to be 35.93 mg/mL, which is 6.3 and 1.96 times higher than the oral administration and transdermal evaporation delivery system without ethanol, respectively, at the same dose.^55^

Santosh *et al.* studied the transport of fentanyl across silicone membranes from subsaturated, saturated and supersaturated residues prepared by employing co-solvent approach (PG:water, 60:40 % v/v) or by employing a volatile solvent (PG: ethanol, 60:40 % v/v). For both type of formulations (PG:water and PG:ethanol), there was a good correlation between the mean flux and theoretical DS. Furthermore, no significant differences were observed in the mean flux between PG:water and PG:ethanol finite dose studies except for the 2DS formulations. Higher permeation enhancement observed with ethanol-based formulations across the DS, which was clearly resulted from an enhanced thermodynamic activity of drug with ethanol evaporation. The only difference between finite and infinite dose studies is the donor reservoir capacity for the drug and solvent.^56^

Santosh *et al.* investigated the role of vehicles/PEs such as PG, OS and IPM on the permeation of fentanyl across human skin *in-vitro* and then a model spray formulations were prepared consisting of 10% (v/v) of individual PEs in ethanol. It was observed that increasing the DS did not promote drug permeation for formulation containing PG but increase of drug permeation observed with formulation containing IPM and OS. This was attributed to the faster permeation of PG from the skin surface compared to IPM and OS. PG and IPM were found to promote drug solubility in the membrane whereas OS acted as PE by increasing drug diffusion in the skin. Furthermore, OS containing formulation significantly improved drug permeation and reduces the lag time compared to IPM and PG.^57^

Intarakumhaeng and Li tried different solvents having different evaporation and penetration property such as ethanol, hexane, isopropanol, and butanol in order to explain their influence on skin deposition/absorption of corticosterone. There were no significant differences in solvent-induced deposition/permeation (i.e., skin permeation due to solvent convection) of corticosterone on/across the SC and also there was no correlation between the rate of absorption of the permeant and the rate of solvent evaporation/penetration was observed. Conversely, for the slow evaporating solvents such as water, PG and PEG 400, there was relationship between permeant absorption and rate of solvent evaporation i.e., slower absorption of corticosterone was evident for the slow evaporating solvent which is due to the change in thermodynamic activity of the permeant during skin absorption.^58^

### Spray systems (MDTS)

Until 2007, aerosols as topical spray were used to deliver the drug across the skin. It is known that aerosol system depends on the power of compressed or liquefied gas to expel the content from the container. Recently, these propellant based sprays have been substituted by propellant free and solvent and polymer based MDTS which can deliver metered amount of drug per actuation. The concept of MDTS was first developed by Victorian college of Pharmacy, Australia and the first commercial product was developed and marketed by Acrux limited.^59,60^ MDTS represents evaporation topical aerosol product composed of drug, solvent systems (volatile:non-volatile), PEs and polymers.^61,62^ Common drugs which are developed as MDTS are estradiol, testosterone, nestrone etc. Methanol, ethanol, dichloromethane, isopropanol are considered as volatile solvent whereas PG, water and diethyl sebacate are used as non-volatile component. In case of polymers, any natural or synthetic polymers can be used. Terpenes, pyrrolidone, azones, alcohol and surfactants are used as PEs.^59,61,63^ In addition, Acrux company developed a lipid like and generally recognized as safe (GRAS) category of PE called as ACROSS® which is added to MDTS® to increase the drug permeation by forming depot system in skin.

**Figure 3:**
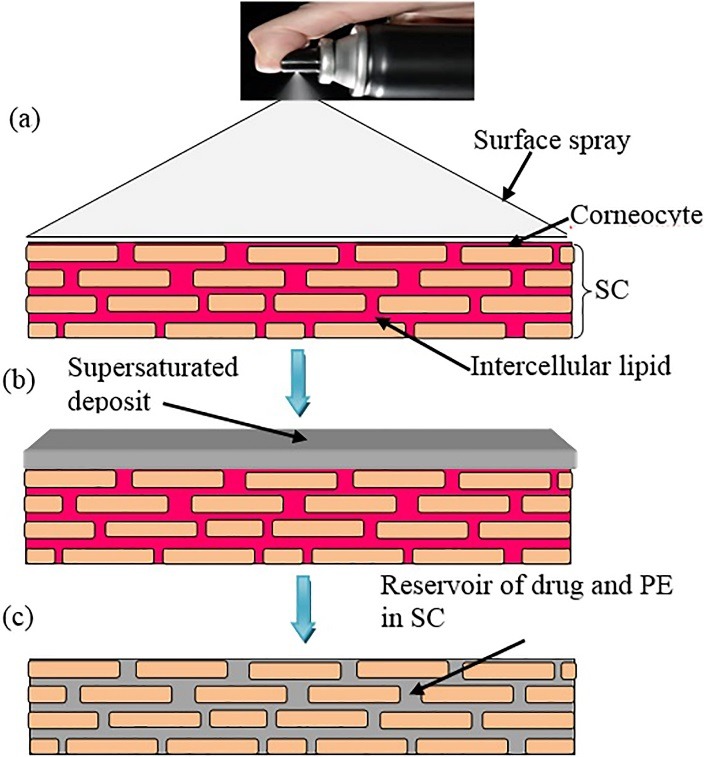
Schematic representation of mechanism of action of MDTS; (a) Surface spray applied to the SC, (b) forced partitioning concept where rapid evaporation of volatile vehicle followed by partitioning of drug and PE into SC, (c) drug and PE form a reservoir within the SC which is lipid in character and water resistant [Modified from Reference 31].

This type of system has more advantages compared to solution and semisolid preparations: (i) as it is a spray system the volume per area of administration can be precisely defined with highly reproducible manner, and (ii) it also helps the formulation to be uniformly distributed on the skin over a defined area after application.^27,64,65^ The major advantage of this system is that it can form drug reservoir/depot in the skin instead of over the skin as in case of patch.^64^ These systems are forming drug reservoir/depot within the SC in step wise manner as illustrated in Figure 3. It is depicted in three steps: (a) actuation of MDTS released desired amount of formulation on to the skin surface, (b) the surface deposit of dosage form, which is shown as pyramid, gradually decreases in its size due to both rapid evaporation of volatile component of the solvent mixture and forced partitioning of drug and PEs into SC, and (C) this partitioning of drug and PEs resulting into formation of their reservoir within the SC and thereby releasing the drug into the systemic circulation in sustained manner. MDTS increases drug diffusivity by SC lipid fluidization or lipid phase.^61^

Leichtnam *et al.* evaluated testosterone permeation across hairless rat skin from a series of ethanol/PG/water formulations to obtain optimal composition followed by its incorporation with modification in to mechanical spray for feasible transdermal spray formulation. Among various combinations, saturated solution of testosterone in 1:1:1 ethanol/PG/water was exhibited highest flux of 1.7±0.2 mg/cm^2^/h. Out of five formulation developed for mechanical spray only 1:1 ethanol/PG showed comparable flux and improved sprayability (due to absence of water). When propellant was added, only 3:1 ethanol/PG was found to be stable and all other formulations either showed phase separation or immediate crystallization. Furthermore, increasing the percentage of ethanol in the mixture increased evaporation rate without any alteration in testosterone permeation.^64^

Leichtnam *et al.* investigated various PEs in order find out suitable combination of PEs, solvent system and/or propellant for the delivery of testosterone from a spray/aerosol. Among the series of PEs used to pre-treat the skin before the application of spray, IPM was found to be most efficient as it increased the penetration enhancement of testosterone by more than 5 times from a solvent system containing 1:1 ethanol/water. The flux was further enhanced with solvent system of ethanol/PG (3:1), which is a more compatible vehicle for the spray. After incorporation of IPM in the range of 10-25% into aerosol system containing 50% v/v propellant, flux was found to be increased only 2.5-fold at highest level of IPM from both the spray and aerosol. The amount of pulverized drug finally contacted the skin surface represented only approximately 10% and 40 % from spray and aerosol, respectively, which was less than expected.^66^

Bakshi *et al.* developed oxybutynin containing metered dose spray formulation for transdermal delivery. A series of combinations of solvent system composed of ethanol/acetone/methylal were prepared and a ratio of 2:1:2 was selected as best combination based on various *in-vitro* parameters. Furthermore, different film forming agents were tried and carbopol and lutrol at 0.5 and 0.1%, respectively, have showed good clarity, spray pattern and evaporation rate. *In-vitro* diffusion study across semipermeable membrane demonstrated that the drug release was almost 59% over a period of 24 h. There was no skin irritation when tested on rabbit. In addition, above formulations were found to be stable.^67^

Terri *et al.* applied novel MDTS of estradiol (1.7%) to healthy postmenopausal women and investigated the steady state pharmacokinetics of estradiol and its metabolites such as estrone and estrone sulfate. The formulation was administered (1.53 mg/spray) once daily for 14 days in three different spray groups (1, 2, and 3) and samples were collected on day 1, before the first dose was administered, to 21 days including 7 days after the last dose administered). It was observed that the steady state was reached by day 7 or 8 for all the three groups and is maintained up to 21 day. The mean eastrdiol and estone concentrations at 14 days following 1, 2, 3-sprays were found to be 36 and 50, 57 and 54, and 54 and 71 pg/mL, respectively. Maximum concentration for estradiol was observed at 18-20 h (t_max_). Finally, it was concluded that MDTS of estradiol produces low serum estron concentration.^68^

Davison *et al.* performed a pilot study on healthy postmenopausal women stabilized on estrogen in order to understand the effect of testosterone therapy on their cognitive performance such as visual and verbal as well as memory. Treatment group of women showed significant effect of testosterone on the cognitive performance in the domain of verbal and memory compared to untreated group.^69^

Lu *et al.* developed MDTS formulations of testosterone as topical solutions composed of volatile vehicle (ethanol) and non-volatile vehicle/PEs such as azone, IPM, PG, and NMP and film forming polymers (FFPs) such as Eudragit® E PO, Eudragit® RL PO, Plasdone® S630 and PVP K30 and evaluated their efficacy. Among the PEs and FFPs used in the permeation study, azone at 9% (v/v) and Plasdone® S630 exhibited highest flux for testosterone. Out of total 9 formulations prepared, formulation containing 10% (w/v) testosterone, 9% (v/v) azone and 91 % (v/v) ethanol showed good skin permeation and acceptable PE content and drug concentration. Furthermore, this optimized formulation was non-irritating in rat model and showed a different plasma-time profile from that of Testopatch® (commercially available product) with Testopatch® exhibited a more sustainable drug release.^27^

Lu *et al.* prepared different MDTS formulations containing dexketoprofen to screen different PEs such as azone, PG, IPM, lauryl lacetate (LA) and FFPs viz. Eudragit RL PO, Plasdone S-630, PVP K30 and Kollidone PF 12. There was no significant difference of drug permeation among all the FFPs. IPM among the PEs was found to be appropriate one and the optimized formulation was containing 7% (w/w) dexketoprofen, 7% (v/v) IPM and 93% (v/v) ethanol. Above formulation showed both anti-inflammatory and anti-nocieptive activities with no sign of skin irritation.^62^

Patel *et al.* developed an user friendly MDTS of Lopinavir to be used in combination of chemical and physical method (microneedle technique) to improve overall penetration enhancement across pig ear skin. Formulation having 5% w/v of Kollidon® showed best volatilization and spreadability property. *Ex-vivo* studies indicated a significantly higher penetration enhancement of 1.77 and steady state transdermal flux of 52.5 µg/cm^2^/h across microporated pig ear skin. Furthermore, *in-vivo* investigation exhibited 3-fold increase in relative bioavailability of lopinavir through transdermal route compared to oral suspension of marketed tablet. In addition the system was found to be safe and stable as well as have potential to be used in clinical settings.^70^

Ranade *et al.* designed topical film-forming metered dose spray formulations of ropivacaine for the management of pain. Permeation study was performed on porcine skin and after the permeation study; skin sample was visualized under conofocal microscopy to ensure the potency of designed formulations. The results demonstrated a high level of drug concentration in the skin layers. The anti-nociceptive efficacy study showed that the formulation has comparable efficacy to the conventional lidocaine gel.^71^

Klose *et al.* prepared spray formulations of antiparkinson’s agent such as ropinirole, pramipixole, sumanirole and cabergoline with aqueous ethanol as evaporating solvent and padimate O/OS as PEs. They observed that the formulations with PEs deliver more antiparkinson’s agent through the shed skin of snake than the corresponding formulation.^72^

Morgan *et al.* successfully developed a topical spray system of oestradiol (1 and 1.7% w/v) and its derivative ethinyl oestradiol for its systemic delivery across the skin of animals and human. Other ingredients present in the system include OS/padimate O as PE and aqueous ethanol as evaporating solvent.^73^

Lulla *et al.* developed a topical spray system that can be sprayed onto the skin to form a stable breathable film or patch over a period of days. The system composed of estradiol and alendronate sodium as active ingredients, eudragit E 100 as a self-adhesive, plastoid B as a film former, PG as a humectant, sodium lauryl sulfate as a solubilizer for the drug, acetone as a volatile solvent for quick drying and non-occlusive vehicle and P11 as a propellant to expel the product out of the container. It was observed that the above composition was able to form stable thin film which was observed up to 24 h after formation and can deliver the drug in a sustained manner from 1 to 5 days.^74^

Lulla and Malhotra patented transdermal spray formulation for testosterone (16.66%). Vinyl pyrrolidone/vinyl acetate (VP/VA) copolymer was used as film forming polymer and ethanol and acetone combination was used as solvent system. The copolymer acted as antinucleating agent at a concentration 1-10%. Above formulation usually forms uniform thin film on the skin within 60 s when sprayed from metered dose spray container at a fixed distance from the skin surface. In addition the above concentration of testosterone in the formulation was able to penetrate the skin and reached to blood circulation in desired concentration.^75^

Watkinson *et al.* patented transdermal spray system for volatile active ingredients such as nicotine and selegiline and volatile solvents selected from ethanol, isopropanol or their mixture in a concentration ranging from 0.5 to 25% by weight to a area of human skin (20 cm^2^). It was claimed to have better potential for the treatment or prophylaxis of Parkinson’s disease and reduced craving for cigarettes in a smoker.^76^

Wan and Watkinson investigated transderrnal spray system of norethisterone acetate, nestorone, estradiol and testosterone containing PEG 200, 400 and OS as PE. They observed; (i) PEG 200 in combination with OS significantly enhance the permeation of norethisterone acetate and estradiol across the human epidermis in vitro, (ii) PEG 400 did not exhibit any significant effect on the permeation of Nestorone whereas it inhibit the permeation of ethylestradiol through human epidermis *in-vitro*, (iii) PEG 200 was significantly enhance the permeation of testosterone across human epidermis *in-vitro* which was not seen when PEG 400 was used as PE, (iv) PEG 200 showed significant increase in permeation enhancement of estradiol.^77^

Morgan *et al.* claimed that a transdermal spray formulation of testosterone comprising of 0.1 to 10% weight of testosterone, 0.1 to 10% weight of one or more PEs consisting of at least OS and padimate O, 85 to 99.8 % by weight of a volatile solvent selected from a group consisting of ethanol, isopropanol and their mixture and optionally a gelling agent. Upon application of the composition, evaporation of solvent at body temperature forms an amorphous deposits leading to enhanced delivery of testosterone across the skin. Similarly, buspirone exhibited maximum permeation across human epidermis when the formulation composed of 95% of ethanol and OS as volatile solvent and PE, respectively. This is because of the formation of amorphous form of the drug leading to both zero and first order kinetics.^78^

**Table 1:** List of evaporative drug delivery systems comprising of drugs, solvent systems and polymers

**Types of formulation**	**Drug**	**Solvent systems (Non volatile/Volatile)**	**Polymer (s)**	**References**
**Solutions and semisolids**	Fluocinolone acetonide and its acetate ester	PG, water and IPM	-	[45]
		PG and IPM/ Isopropanol	-	[46]
	Hydrocortisone	Water/Acetone	-	[47]
	Nifedipine	IPM and PG/ Acetone	-	[48]
		Diethyl sabacate/ Ethanol	-	[49
		PG and IPM/ Acetone	-	[50]
	Minoxidil	PG and water/ Ethanol	-	[51]
	Nifedipine	Butanone and water/ Ethanol	-	[52]
	Piroxicam	PG and water	HPMC	[20]
		-	-	[38]
	Ketoprofen	PG/ Octanol, ethanol and OA	-	[34]
	Sodium nonivamide acetate	Water/ Ethanol	-	[53]
	Ibuprofen	PG and water	MC and HPC	[29]
	Lavendustin derivative	PG, PEG, IPM and silicone oil/ Ethanol	HPMC and CD	[54]
	Oxybutynin	PG and OS/ ethanol	-	[35]
	Praziquantel	EGPE, tetrahydrofuran, 1, 4-dioxane, oleic acid and DMSO/ Ethanol	-	[55]
	Fentanyl	PG and water/ Ethanol	-	[56]
		PG, OS and IPM/ Ethanol	-	[57]
	Corticosterone	Water, PG and PEG 400/ Ethanol, hexane, isopropanol, and butanol	-	[58]
**Sprays**	Dexketoprofen	Azone, PG, IPM and LA/ Ethanol	Eudragit RL PO, Plasdone S-630, PVP K30 and Kollidone PF 12	[62]
	Testosterone	PG and water/ Ethanol	-	[64]
		IPM and water/ Ethanol	-	[66]
	Oxybutynin	-/ Ethanol, acetone, methylal	Carbopol and lutrol	[67]
	Estradiol	-	-	[68]
	Testosterone	-	-	[69]
		Azone, IPM, PG and NMP/ Ethanol	Eudragit® E PO, Eudragit® RL PO, Plasdone® S630 and PVP K30	[27]
	Lopinavir	-	Kollidon®	[70]
	Ropivacaine	-	-	[71]
	Ropinirole, pramipixole, sumanirole and cabergoline	Padimate O and OS/ Ethanol	-	[72]
	Oestradiol and ethinyl oestradiol	Padimate O and OS/ Ethanol	-	[73]
	Estradiol and alendronate sodium	PG/ Acetone	Eudragit E 100 a self adhesive, plastoid B	[74]
	Testosterone	-/ Ethanol and acetone	VA/VP	[75]
	Nicotine and selegiline	-/ Ethanol, isopropanol	-	[76]
	Norethisterone acetate, nestorone, estradiol and testosterone	PEG 200, 400 and OS/ -	-	[77]
	Testosterone and buspirone	OS and padimate O/ Ethanol, isopropanol	-	[78]

## Commercial products

Acrux Pvt. Ltd. is the first company to commercialize MDTS with lunching number of products in market such as Evamist®, Lenzetto®, Ellavie®, Axiron®, Nestorone®. These products provide fast-drying, invisible sprays or liquids with outmost cosmetic acceptability. Moreover the other advantages of above system are attainment of steady state plasma concentration for four days after a single application and very low skin irritation rate because of non-occlusive delivery.^79,80^A list of commercial products based on evaporative principles with their brand name, marketing company, active ingredient, formulation type and use are presented in Table 2.

**Table 2:** List of commercial products based on evaporative principles

**Product (Brand name)**	**Marketing company**	**Active ingredient**	**Formulation type**	**Purpose**
Evamist®	Perrigo (From 2014)	Estradiol	Spray (MDTS)	Menopause symptoms related to vasomotor and hot flushes
	KV Pharmaceuticals (from 2007 to 2014)			
Lenzetto®	Gedeon Richter			
Ellavie®	Aspen Pharmacare and Vifor pharma			
Axiron®	Eli Lilly	Testosterone	Solution	Hypogonadism or low/no testosterone
Nestorone (NES) MDTS®	Acrux Pvt. Ltd. (Phase-I trial completed) partnered with Population Council and Fempharm Pvt. Ltd	Nestorone	Spray (MDTS)	Contraception
Nicotine MDTS®	Acrux Pvt. Ltd. (Phase-I trial completed)	Nicotine	Spray (MDTS)	Smoking cessation
Recuvyra®	Elanco Pvt. Ltd.	Fentanyl	Spray (MDTS)	Postoperative pain (for veterinary use)

## Evamist®, Lenzetto®, Ellavie® (Estradiol transdermal spray)

Estradiol spray system was the first MDTS developed by Acrux Pvt. Ltd. to treat menopause symptoms such as severe hot flushes and it was approved by FDA in three brand names such as Evamist, Lenzetto® and Ellavie®. These were licensed to different companies to market it all across the globe. In 2007, Evamist® licensed to KV Pharmaceuticals to market it in US, but latter on it was distributed by Perrigo from 2014 as the former company was acquired by the latter. Gedeon Richter received multiple marketing approvals for estradiol spray and in 2016 it was launched in Europe and Eurasia in the brand name of Lenzetto®. Ellavie® is a unique estradiol skin spray which is used to treat vasomotor symptoms associated with menopause. From 2009 Ellavie® was marketed and distributed in Australia by Aspen Pharmacare and in Switzerland by Vifor pharma.^81^

**Figure 4:**
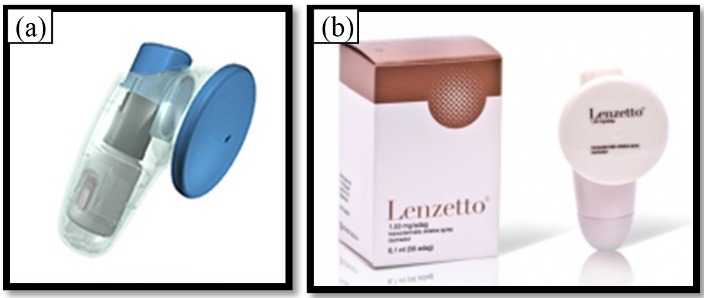
Commercial product of estradiol: (a) Evamist^®^ and (b) Lenzetto^®^

All the above products are small and hand-held spray (Figure 4), design to deliver a predetermined dose of estradiol in the systemic circulation on sustained basis over 24 h across the skin. They are placed gently against the skin following which an actuator button is pressed to release light spray containing estradiol. The spray is fast drying thereby leaving an invisible film after application.^31,82^ When measured by radioimmunoassay after estradiol-MDTS post menopausal women have shown higher plasma level of estradiol than baseline value.^63^

### Axiron® (Testosterone solution)

**Figure 5:**
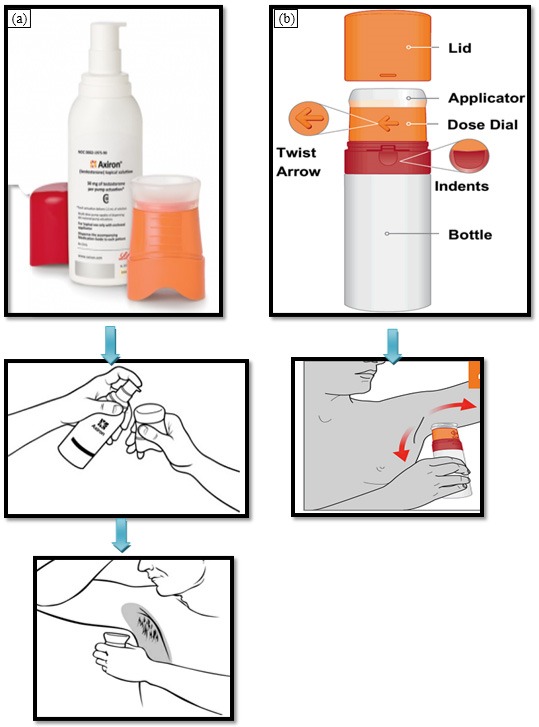
Commercial product of testosterone (Axiron®) and their way of application; (a) pump actuated, and (b) twist actuated

Testosterone topical solution was marketed in the brand name of Axiron®. It was licenced to Eli Lilly and was launched by the same company in the countries such as US, Canada, Australia Germany, Braziland South Korea. Axiron® is used to treat adult males who are suffering from hypogonadism or low/no testosterone. It is available in two forms; pump actuated (Figure 5a) and twist actuated (Figure 5b) metered dose pump and are approved for application with the help of an armpit (underarm) applicator.

Axiron® is consisting of three parts; pump, cap and applicator cup as soon as in Figure 5. Initially, the cap and the applicator cup are to be removed from the pump. Then, the nozzle of the pump is to positioned over the applicator and was pressed gently to release preset dose of testosterone (30, 60, 90, and 120 mg) on to the applicator. This is followed by the rubbing action (steady up and down movement) into the armpit application site. In case of twist actuated (Figure 5b), the applicator is fixed on the bottle. Therefore, it is directly rubbed on to the patient’s armpit. The application site is to allow for drying for three minutes before wearing a dress.^83^

Acrux Pvt. Ltd. is also developing a fast drying testosterone MD-Lotion® which can be applied directly into the patient’s armpit using an innovative “no-touch” applicator, in similar way as deodorant. In 2015, Elli Lily successfully completed phase III trial on the product in different countries over the world. The topical solution can only be applied by touching the applicator to the armpit. This necessitates an extra step of washing and drying of applicator prior to next use. In this context “no-touch” applicator is having a clear advantage.^84^

### Nestorone (NES) MDTS® (Nestorone transdermal spray)

**Figure 6:**
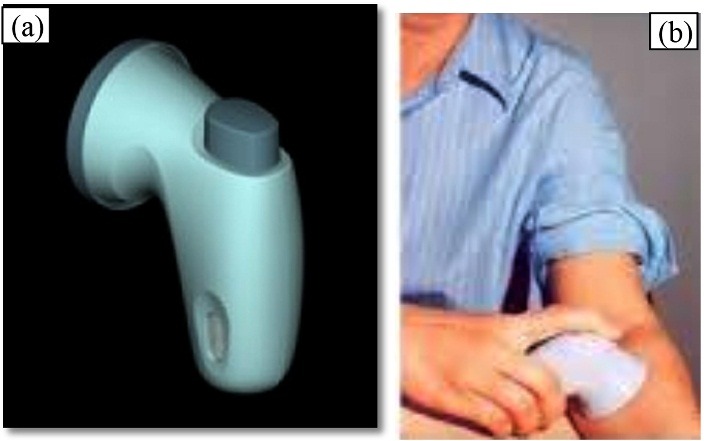
Commercial product of Nestorone; (a) nestorone MDTS®, and (b) its application

Nestorone is a non-androgenic novel progestin used for non-oral contraception. Acrux Pvt. Ltd. developed a progestin-only-spray-on-contraceptive MDTS for Nestorone (Figure 6 (a) and (b)). Nestorone spray is found to be highly active transdermally resulting in good systemic bioavailability.^85,86^ Fraser *et al.* reported that serum levels of Nestorone was achieved within the range of 285-290 pmol/L and was able to block ovulation in case of 83% of the women taken the spray during the study. This result proven that the above serum concentration of the drug is sufficient enough to provide effective contraception.^87^ Thereafter, Acrux Pvt. Ltd. partnered with Population Council and Fempharm Pvt. Ltd. to develop a MDTS spray system containing both Nestorone and an estrogen or 17-ethinylestradiol in order to explore the possibility of delivering once daily dose to illicit contraception. The product is under phase-1 trial and was found to be releasing the drug into blood circulation over a period of 72 h. The current status is that the company involved in the development process abandoned the project.^88^

### Nicotine MDTS® and Fentanyl MDTS®

In 2007, Acrux Pvt. Ltd. has announced that Nicotine MDTS®, a newly developed transdermal spray system for the cessation of smoking, is added to the pipeline. The company successfully completed phase I clinical trial of three different spray formulations of nicotine on sixteen subjects in Australia. Similarly, Acrux Pvt. Ltd. also developed fentanyl MDTS® to treat chronic pain and given distribution rights to CSL Ltd.

**Figure 7:**
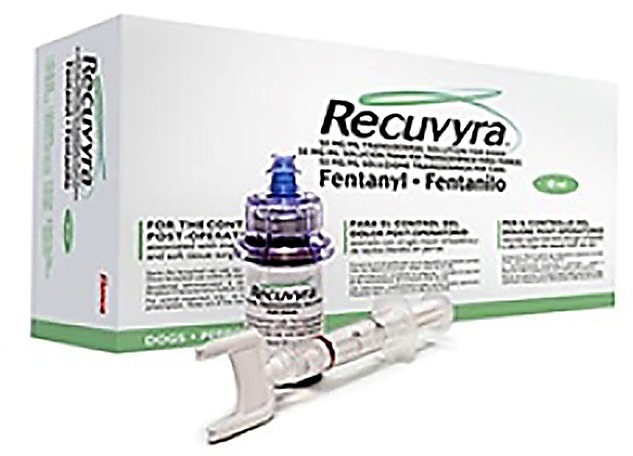
Commercial fentanyl transdermal solution for veterinary use (Recuvyra®).

Apart from above MDTS® developed for human use, Acrux Pvt. Ltd. also developed transdermal fentanyl solution Recuvyra®, a veterinary product, used for the control of postoperative pain associated with major orthopaedic and soft tissue surgery (Figure 7). It is first of kind of innovative evaporative products (50mg/ml) lunched by Elanco Pvt. Ltd. to market it globally and has the ability to control pain for 4 days in a single dose.

## Discussion

Drug delivery through transdermal route seems to be very attractive concept, but not all classes of drugs will be able to permeate the SC barrier in sufficient amount that can illicit the therapeutic action. The physico-chemical properties which are important for a drug to permeate the barrier layer of skin by passive diffusion includes small molecular size (less than 500Da), log partition coefficient preferentially within range of 1 to 3.5, aqueous solubility of greater than 100 µg/mL, low drug dose suitably less than 10mg/day and melting point less than 200°C.^21,89^ The individual drugs suitable for TDD are those which undergoes first pass metabolism either in liver of GI tract, causing irritation in the stomach, elimination half life less than 3h and showing absorption window in oral route. At present, no such drug molecules are approved by USFDA which possess all above properties. However, it is possible only when the design and development of drug molecules with maximum number of desired properties to suit TDDD will be developed.

In these circumstances evaporation TDDS composed of only two component system can be explored to a maximum extent to include more drugs in to the fold of TDD route. The concept involved in evaporation system is supersaturation. The volatile component of the solvent mixture evaporates leaving behind supersaturated solution in non-volatile solvent component on the skin surface after application. The non-volatile component in which the drug is soluble thereby preventing the drug precipitation. This resulted in partitioning of drug across the skin in higher amount that can provide required drug concentration in systemic circulation.^31^

These systems can be formulated into various types of formulations such as solutions, semisolids, and sprays. The MDT spray systems have the added advantages of delivering the drug in metered manner that can provide precise dose. In addition these systems are able to deliver the dose at the site of action in case of topical drug delivery. There are many commercial products containing number of drugs such as Evamist®, Lenzetto®, Axiron®, Netorone MDTS®, Recuvyra®, and Nicotene MDTS® are already available and there are many drugs in the pipeline.

## Conclusion

Despite of numerous constraints, drug delivery through transdermal route continues to grow and expand because of the development of new technologies such as the use of sonophoresis, electroporation, microneedle, chemical PEs and novel supersaturation concept. This technological advancement in TDD sector led to successful delivery of drugs ranging from potent (low dose) to moderately potent drug. Among all, supersaturation concept is standing tall as it has the flexibility of delivering the dose in solution, semisolid or in the spray form. In addition the drug dose can be precisely and accurately administered with MDTS which avoids the intra- and inter-patient dose variation. In this context, Aptar group taken a step ahead in developing metered dose spray pumps and dispenser for topical and transdermal products which can be combined with MDTS to achieve dial-a-dose of transdermal delivery with highest degree of accuracy and precision.^90^

Like other techniques, this concept is totally not free of disadvantages. The main issue remains is the stability of supersaturated formulations as with the increase in drug concentration there are more probability of drug crystallization. But to a large extent the problem is minimized by adding antinucleating excipients such as polymers. As of now the evaporative technique using supersaturated concept is restricted to drugs of fewer class e.g., hormones, anti-asthmatics, NSAIDs, central analgesics and drug addiction. But the positive outcome of this technique is that there are many formulations in the market and many are in the pipeline, particularly as MDTS. We anticipate more and more classes of drugs will be delivered using this technique and will be available in the market. We also hope that evaporative technique along with other TDD technique will improve health-related quality of life of patient globally by preventing, diagnosing and controlling the diseases.

## Conflict of Interest

The author declares that this article content has no conflict of interest

## Ethical Issues

Not applicable.
